# Comparative Characterization of Osteoclasts Derived From Murine Bone Marrow Macrophages and RAW 264.7 Cells Using Quantitative Proteomics

**DOI:** 10.1002/jbm4.10058

**Published:** 2018-07-07

**Authors:** Andrew YH Ng, Chengjian Tu, Shichen Shen, Ding Xu, Merry J Oursler, Jun Qu, Shuying Yang

**Affiliations:** ^1^ Department of Anatomy and Cell Biology School of Dental Medicine University of Pennsylvania Philadelphia PA USA; ^2^ Department of Oral Biology School of Dental Medicine University at Buffalo Buffalo NY USA; ^3^ New York State Center of Excellence in Bioinformatics and Life Sciences Buffalo NY USA; ^4^ Department of Pharmaceutical Sciences School of Pharmacy and Pharmaceutical Sciences University at Buffalo NY USA; ^5^ Division of Endocrinology Metabolism, Nutrition, and Diabetes Mayo Clinic Rochester MN USA

**Keywords:** OSTEOCLASTS, PROTEOMICS, BONE MARROW MACROPHAGES, RAW 264.7 MODELS

## Abstract

Osteoclasts are bone‐resorbing cells differentiated from macrophage/monocyte precursors in response to macrophage colony‐stimulating factor (M‐CSF) and receptor activator of NF‐κB ligand (RANKL). *In vitro* models are principally based on primary bone marrow macrophages (BMMs), but RAW 264.7 cells are frequently used because they are widely available, easy to culture, and more amenable to genetic manipulation than primary cells. Increasing evidence, however, has shown that the vastly different origins of these two cell types may have important effects on cell behavior. In particular, M‐CSF is a prerequisite for the differentiation of BMMs, by promoting survival and proliferation and priming the cells for RANKL induction. RAW 264.7 cells readily form osteoclasts in the presence of RANKL, but M‐CSF is not required. Based on these key differences, we sought to understand their functional implications and how it might affect osteoclast differentiation and related signaling pathways. Using a robust and high‐throughput proteomics strategy, we quantified the global protein changes in osteoclasts derived from BMMs and RAW 264.7 cells at 1, 3, and 5 days of differentiation. Data are available via ProteomeXchange with the identifier PXD009610. Correlation analysis of the proteomes demonstrated low concordance between the two cell types (*R^2^* ≈ 0.13). Bioinformatics analysis indicate that RANKL‐dependent signaling was intact in RAW 264.7 cells, but biological processes known to be dependent on M‐CSF were significantly different, including cell cycle control, cytoskeletal organization, and apoptosis. RAW 264.7 cells exhibited constitutive activation of Erk and Akt that was dependent on the activity of Abelson tyrosine kinase, and the timing of Erk and Akt activation was significantly different between BMMs and RAW 264.7 cells. Our findings provide the first evidence for major discrepancies between BMMs and RAW 264.7 cells, indicating that careful consideration is needed when using the RAW 264.7 cell line for studying M‐CSF‐dependent signaling and functions. © 2018 American Society for Bone and Mineral Research. © 2018 The Authors. *JBMR Plus* published by Wiley Periodicals, Inc. on behalf of American Society for Bone and Mineral Research.

## Introduction

Osteoclasts (OCs) are the cells uniquely responsible for resorbing bone and play important roles in both physiological and pathological bone remodeling.^1^, ^2^, ^3^ In the endeavor to discover new and better treatment strategies against osteoporosis, much effort has been put into elucidating the critical signaling pathways that regulate OC differentiation. In the presence of macrophage colony‐stimulating factor (M‐CSF) and receptor activator of NF‐κB ligand (RANKL), the two cytokines necessary and sufficient for OC differentiation, precursor cells of the hematopoietic lineage undergo dramatic reconfiguration where they fuse to form expansive, multinucleated OCs. These cells adhere and secrete acids and hydrolases onto the bone surface to resorb bone.^1^, ^2^, ^4^

Both bone marrow macrophages (BMMs) and RAW 264.7 cells have the potential to differentiate into OCs, but the vastly different origins of these two cell types may have profound effects on cell signaling and behavior. While BMMs are primary cells most frequently isolated from the marrow cavity of rodent long bones,^5^ RAW264.7 cells are a transformed macrophage‐like cell line derived from the lymphoma of a male BALB/c mouse infected by the Abelson murine leukemia virus (A‐muLV).^6^, ^7^ The retrovirus encodes an oncogenic form of the Abelson kinase, v‐Abl, which is a fusion protein where portions of the retroviral Gag protein substitute regions of the SH3 domain of c‐Abl, rendering the tyrosine kinase constitutively active.^8^, ^9^, ^10^, ^11^, ^12^ Studies have shown that the expression of v‐Abl leads to the constitutive activation of mitogenic signaling pathways (e.g. Ras, Jak‐Stat, JNK, and PI3K), resulting in uncontrolled cell proliferation—a key feature of cell transformation.^11^ In human patients, the fusion protein composed of the breakpoint cluster region (BCR) and ABL1 (BCR‐ABL) causes chronic myeloid leukemia.^13^ Patients receiving inhibitors to c‐Abl often show reduced bone turnover because of impaired osteoblast (OB) and OC function.^14^ These inhibitors can directly suppress OC formation and bone resorption through various mechanisms, including preventing M‐CSF‐induced phosphorylation of c‐Fms.^15^, ^16^ Additionally, BMMs prepared from c‐Abl null mice show impaired OC differentiation and reduced TRAP activity.^17^ c‐Abl undoubtedly plays an important role in OCs, but how the constitutive activity of v‐Abl affects the behavior of OCs derived from RAW 264.7 cells is unclear.

The osteoclastogenic potential of RAW264.7 cells was first demonstrated by Hsu and colleagues,^18^ who found that RAW 264.7 cells expressed RANK and could readily generate large, TRAP^+^ cells capable of resorbing bone in the presence of RANKL. RAW 264.7 cells have since been an important cell line for bone research because of their widespread availability, homogeneous nature of pre‐OC population (devoid of osteoblasts, lymphocytes, stroma, etc.), and ease of culture and transfection for genetic manipulation.^19^ The significance of RAW 264.7 cells is evidenced by the >530 publications to date that have employed this cell model in OC studies (PubMed). Some among these studies sought to characterize the OCs derived from RAW 264.7 cells and have largely determined that these OCs do indeed mimic their BM‐derived counterpart, although some irregularities have raised awareness of fundamental problems that have yet to be rectified.^20^, ^21^ Notably, multiple reports have documented that RAW 264.7 cells can proliferate and undergo OC differentiation independently of M‐CSF.^22^, ^23^, ^24^, ^25^ One study in particular demonstrated that RANKL augments M‐CSF production in RAW 264.7 cells, which helps to explain why exogenous M‐CSF is not needed.^21^ Following these findings, we further investigated the molecular differences between RAW 264.7 cells and BMMs in OC differentiation and its related signaling pathways.

Proteomics is a powerful tool that has led to numerous discoveries of proteins and biological processes that drive OC differentiation.^26^ Notably, this technique was recently used to map the podosome proteome and helped to advance our understanding of determinants in the macrophage multinucleation process.^27^, ^28^ In other examples, proteomics analysis identified changes in the expression of proteins involved in metabolism and redirection of energy flow toward bone resorption in OCs.^29^ Proteomics can therefore provide a broad yet informative overview of the systemic changes in the differentiating OC. We employed a robust and high‐throughput liquid‐chromatography mass spectrometry (LC‐MS)‐based proteomics approach to characterize the global protein changes of OCs at different stages of the reprogramming process. Our findings provide the first evidence that BMMs and RAW 264.7 cells are significantly different, especially in processes related to cell cycle control, cytoskeletal organization, and apoptosis. These results warrant careful consideration when using the RAW 264.7 cell line for studying M‐CSF‐dependent signaling and functions.

## Materials And Methods

### Materials

The pGEX‐4T‐1‐rRANKL vector was a generous gift from Dr. Steven Teitelbaum that encodes the GST fusion construct containing the biologically active extracellular domain of RANKL (GST‐RANKL).^30^, ^31^ The plasmid was transformed into and expressed in Origami B(DE3) cells (EMD Millipore, Billerica, MA, USA) that co‐expresses chaperone proteins and was generously provided by Dr. Ding Xu. The GST‐RANKL fusion protein was purified using a BioScale Mini Profinity GST cartridge (Bio‐Rad, Hercules, CA, USA) and a subsequent size‐exclusion chromatography step. Endotoxins were removed using the Pierce High Capacity Endotoxin Removal Resin (Thermo Fisher Scientific, Waltham, MA, USA). GNF‐2 was obtained from Selleck Chemicals (Houston, TX, USA).

### Isolation of primary bone marrow cells

Bone marrow cells were obtained from the tibia and femur of 8‐week‐old C57BL/6J mice as described previously.^32^ All studies and procedures performed on mice were approved by the University at Buffalo Institutional Animal Care and Use Committee.

### Cell culture and osteoclast differentiation

The RAW 264.7 cell line was obtained from the American Type Culture Collection (ATCC, Manassas, VA, USA). RAW 264.7 cells and BMMs were cultured in αMEM containing 10% fetal bovine. BMMs and RAW 264.7 cells were treated with M‐CSF and RANKL for 0, 1, 3, and 5 days. The method for generating OCs in vitro from BMMs was described previously.^5^

### Reverse transcription and quantitative PCR

Total RNA was isolated from cells using Trizol reagent (Invitrogen, Carlsbad, CA, USA) following manufacturer's instructions. cDNA was reverse transcribed from 1 µg total RNA using the RNA to cDNA EcoDry Premix Kit (Clontech, Mountain View, CA, USA). Primer sequences were designed using Primer BLAST^33^ and purchased from Integrated DNA Technologies (San Diego, CA, USA). The primer sequences are listed in Supplemental Table S1. All PCR reactions were normalized to the housekeeping gene *Hprt*.^34^ qPCR analysis was performed using the 2× SYBR Green qPCR Master Mix (Bimake, Houston, TX, USA) according to manufacturer's instructions. Data analysis of gene expression was performed using the CFX Maestro software (Bio‐Rad).

### Protein extraction and precipitation/on‐pellet digestion

Cells were harvested using ice‐cold lysis buffer (50 mM Tris‐formic acid, 150 mM NaCl, 0.5% sodium deoxycholate, 1% SDS, 2% NP‐40, pH 8.0) with protease inhibitor (cOmplete, Mini, EDTA‐free; Roche, Mannheim, Germany). Samples were prepared for MS analysis using a previously established method.^35^

### Liquid chromatography‐tandem mass spectrometry analysis

The IonStar LC‐MS experimental pipeline was developed and optimized in a previous study.^(35)^ Details can be found in the Supplemental Methods. A stringent set of criteria including a low peptide and protein false discovery rate (FDR) of <1% and ≥2 peptides per protein was used for protein identification. An ion current‐based quantification method (IonStar processing pipeline) was described previously.^35^ The mass spectrometry proteomics data have been deposited to the ProteomeXchange Consortium via the Proteomics Identifications (PRIDE)^36^ partner repository with the data set identifier PXD009610.

### Bioinformatics analysis

Ingenuity Pathway Analysis (Qiagen, Redwood City, CA, USA) and InnateDB^37^ were used to perform gene ontology (GO) term analysis. Hierarchical clustering analysis and heat map visualization were performed using the *heatplot* function in R Package *made4*. Fuzzy C‐means clustering was performed using the *cmeans* function in R Package *e1071*, and the results were visualized using the R package *ggplot2* and *viridis* color palette.

### Western blot

Cells were cultured in 6‐well plates in complete medium and pretreated with GNF‐2 in serum‐free medium for 3 hours before induction with either M‐CSF (100 ng/mL) or RANKL (200 ng/mL) for the indicated times. BMMs and RAW 264.7 cells were treated with 2 µM and 5 µM GNF‐2, respectively. Western blot was performed as described previously.^38^ The primary antibodies used in this study were as follows: rabbit polyclonal phosphorylated Akt (Thr308) (1:1000, Cell Signaling Technology, Danvers, MA, USA), rabbit monoclonal Akt (pan) (1:1000, CST), rabbit polyclonal Erk1 (Thr202/Tyr204) + Erk2 (Thr186/Tyr187) (1:100, Abcam, Cambridge, MA, USA), rabbit polyclonal Erk1/2 (1:1000, CST), and rabbit monoclonal Gapdh (1:2000, CST). Densitomety analysis was performed using ImageJ^39^ and normalized to the Gapdh intensity. Relative phosphorylation of Akt and Erk was presented as the ratio between the phosphorylated normalized to the nonphosphorylated/total protein.

### Statistical analysis

The proteomics data are representative of three biological replicates (mice) for BMMs and five replicates for RAW 264.7 cells, for each time point of OC differentiation (days 0, 1, 3, and 5). Fold‐change values at each time point were normalized to day 0 (uninduced) and analyzed by two‐tailed Student's *t* test. Proteins demonstrating >0.5 log_2_(fold‐change) and *p* values <0.05 were considered statistically significant. Quantitative data are presented as means ± SD from at least three independent experiments and were analyzed using two‐tailed Student's *t* test. A *p* value <0.05 was considered statistically significant.

## Results

### Characterization of the temporal proteome during OC differentiation

**Figure 1:**
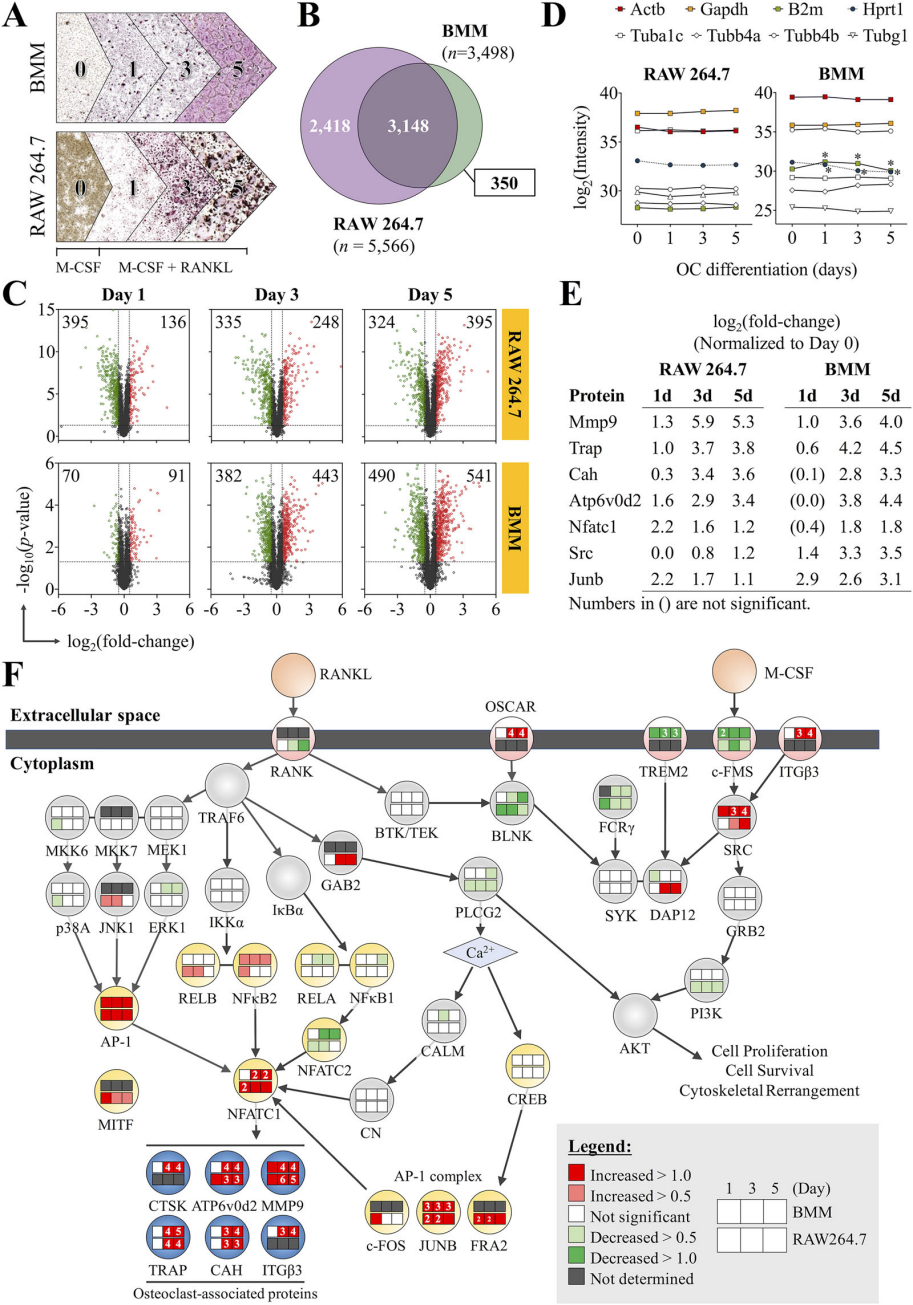
High‐throughput proteomics analysis accurately maps the global protein changes during OC differentiation. (*A*) Proteomics experimental design. The proteomics data are representative of *n* = 3 biological replicates (mice) for BMMs and *n* = 5 replicates for RAW 264.7 cells. (*B*) Venn diagram summarizing the proteins that overlap between the RAW264.7 and BMM data sets. (*C*) Protein expression changes in RAW 264.7 cells and BMMs during OC development relative to day 0. Cut‐offs using *p *< 0.05 and log_2_‐transformed ratio of >0.5 were applied to determine significantly altered proteins. Numbers in the corners of each quadrant indicate the number of proteins that were upregulated (red) or downregulated (green). (*D*) Relative quantification of common internal control proteins at different time points of OC differentiation to demonstrate equivalence of protein amounts in each group. (*E*) The expression of OC marker proteins relative to day 0. (*F*) Summary of canonical pathways activated during OC differentiation. Data are means ± SD. Statistical analysis was performed using Student's *t* test for each time point against day 0 (*p < 0.05). GO = gene ontology.

Proteomics analysis identified 3498 and 5566 quantifiable proteins in the BM‐ and RAW264.7‐derived OCs, respectively, with no missing data at all time points (1, 3, and 5 days after RANKL induction) (Fig. 1 *A*, Supplemental Table S2). A total of 3148 proteins were shared between both data sets, thereby allowing direct comparison of the protein expression levels (Fig. 1 *B*). Within this shared data set of proteins, we identified 1614 unique (non‐redundant) proteins that were significantly altered by M‐CSF/RANKL in at least one time point relative to day 0. Proteins are considered significantly altered if they exceed the thresholds set at *p* < 0.05 and >0.5 log_2_‐transformed ratio (Fig. 1 *C*). To determine whether the total protein quantities across the time points were equivalent, we assessed the relative abundance of housekeeping proteins traditionally used as western blotting internal controls (Fig. 1 *D*). A previous study by Stephens and colleagues examined the stability of a set of housekeeping genes, including β‐actin (*Actb*), glyceraldehyde 3‐phosphate dehydrogenase (*Gapdh*), β2‐microglobulin (*B2m*), and hypoxanthine‐guanine phosphoribosyltrasnferase (*Hprt*) by qRT‐PCR. They determined that *B2m* showed the least variability, whereas *Gapdh* showed the highest variability in BM‐derived OCs.^34^ When using the same criteria for determining significantly altered proteins in the full data set, we found that no housekeeping proteins with the exception to B2m and Hprt were significantly altered relative to day 0. In BMMs, B2m was transiently upregulated, whereas Hprt was downregulated during OC differentiation; none of these proteins, however, were significantly altered in RAW264.7 cells. Because numerous studies have identified major discrepancies in the correlation between mRNA and protein expression levels,^29^, ^40^, ^41^ we were interested in whether housekeeping proteins exhibited any differences in OCs. In contrast to the findings by Stephens and colleagues, we observed that Gapdh levels were consistent throughout OC differentiation in BMMs, whereas B2m and Hprt were not, indicating that these two proteins may not be reliable internal controls for protein samples. In any case, the stability of common housekeeping proteins such as Actb, Gapdh, α‐ (Tuba1c), β‐ (Tubb4a and Tubb4b), and γ‐tubulins (Tubg1) in our data set indicates low intergroup variation and reinforces the reliability of the proteomics data.

To validate the biological relevance of our proteomics data, we focused on proteins belonging to the canonical signaling pathways induced by RANKL and M‐CSF, including the MAPK, NF‐κB, PI3K, calcium signaling, and costimulatory path ways (Fig. 1 *E*, *F*). OC marker proteins, including Cathepsin K (Ctsk), ATPase H^+^ transporting V0 subunit D2 (Atp6v0d2), metalloproteinase 9 (Mmp9), tartrate‐resistant acid phosphatase (Trap), carbonic anhydrase (Cah), and integrin β3 (Itgb3) were dramatically upregulated. The intensities of protein expression were comparable between OCs derived from BMMs and RAW264.7 cells, with the exception of Mmp9, which was more strongly induced in RAW264.7 cells. NFATc1, the master regulator of OC differentiation, was also strongly upregulated; however, BMMs and RAW 264.7 cells demonstrated slightly different kinetics of the protein's expression. In BMMs, NFATc1 upregulation was only significantly starting at day 3, which was maintained through day 5 (+1.8 and +1.8, respectively). In RAW264.7 cells, the peak of NFATc1 expression was at day 1 (+2.2), which gradually decreased at day 3 and day 5 (+1.6 and +1.2, respectively). Interestingly, the different patterns of NFATc1 expression did not affect the expression pattern of the OC marker proteins, which were very similar between the two cell types. In addition to NFATc1, RANKL activates other transcription factors important for OC differentiation, including PU.1, c‐Fos, NF‐κB1 (canonical NF‐κB) and PPARγ, which are known to be activated during the early phase of OC differentiation.^42^ The proteomics data showed that c‐Fos expression was upregulated (+1.0) at day 1 but not at days 3 and 5. The other early factors, PU.1 and NF‐κB1, did not show any upregulation at any of the time points (Fig. 1 *F*). This observation outlines a constraint in our experimental design, such that our data do not cover the early phase response (<24 hours). On the other hand, NFATc1, MITF, and NF‐κB2 (noncanonical NF‐κB), which are known to function during the later phase of OC differentiation, were strongly upregulated at the time points captured in our experiment (Fig. 1 *E*, *F*). Therefore, our proteomics data provide an excellent coverage of signaling pathways important for OC differentiation.

### Unsupervised clustering and correlation analysis reveals major differences in the proteomes of BMMs and RAW264.7 cells in the later phases of osteoclastogenesis

**Figure 2:**
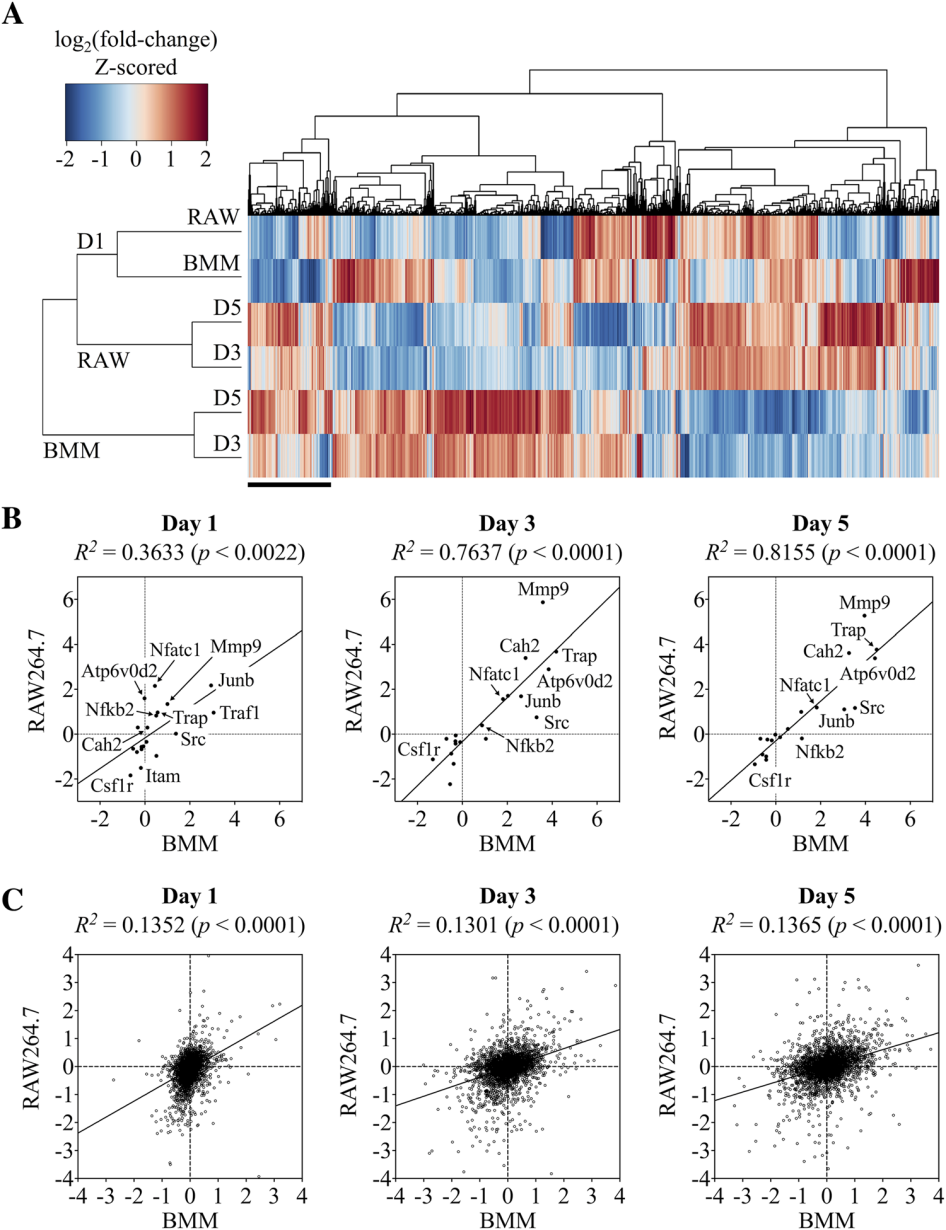
Unsupervised hierarchical clustering and correlation analysis reveals major differences in the proteomes of BMMs and RAW264.7 cells. (*A*) Protein expression changes in BMMs and RAW264.7 cells differentiated using M‐CSF and RANKL for 1, 3, or 5 days. Heat map of all identified proteins (*n* = 3148) analyzed by hierarchical clustering analysis. (*B*) Pearson correlation analysis of OC differentiation marker protein expression (*n* = 20) in RAW264.7‐ and BMM‐derived osteoclasts show a strong positive correlation between the two cell models. (*C*) The same analysis applied to the entire data set (*n* = 3148), however, revealed a relatively poor correlation (*R^2^* ≈ 1.3). Expression values are log_2_‐transformed ratios relative to day 0.

To analyze the concordance between the proteome of OCs derived from BMMs or RAW264.7 cells, hierarchical clustering analysis was performed on all proteins shared between both data sets (*n* = 3148) (Fig. 2 *A*). In general, proteins that were significantly altered at day 3 maintained the same direction of change (up‐ or downregulation) into day 5, but the changes tend to become more pronounced. This observation indicates that the OC progenitors were fully committed by day 3 and that the same transcriptional program was maintained for the remainder of the differentiation process. When assessing how the proteomes of RAW 264.7 cells and BMMs are related, the predominant clustering pattern was based on cell type, followed by the timing of OC differentiation (Fig. 2 *A*). The expression profiles for day 1 BMMs and RAW264.7 cells showed a moderate degree of similarity, demonstrated by their proximity in the left dendrogram and concordance in the heat map. At days 3 and 5, however, the expression profiles for BMMs and RAW 264.7 cells significantly diverged. A subset of proteins at days 3 and 5 exhibited a high degree of concordance (Fig. 2 *A*, black bar), but surprisingly, most proteins showed a weak relationship between the two cell types. Overall, our observations suggest that RAW 264.7 cells and BMMs undergo similar processes during early OC differentiation but may have functional differences during the later phases of OC differentiation.

We performed a correlation analysis to quantitatively determine the similarity between the expression profiles of BMMs and RAW 264.7 cells during OC differentiation. To establish the experimental “upper limit” for which the highest correlation could be achieved, we selected 20 OC markers for correlation analysis that included proteins indispensable for OC formation, including nuclear NFATc1, Mmp9, Atp6v0d2, etc. (Fig. 2 *B*). Given that both BMMs and RAW264.7 cells readily differentiate into OCs, we expected OC markers to be induced similarly despite any potential differences between these two cell types. As expected, the expression of OC markers was generally proportionate in both cell types, with the exception to Mmp9 and Src. Mmp9 had a more prominent increase in RAW264.7 cells (+5.6) compared to BM cells (+3.8) at days 3 and 5. On the other hand, Src in RAW264.7 cells only showed a moderate induction (+1.0) compared with BM‐derived OCs (+2.4). Correlation coefficients (*R^2^*) calculated using the Pearson method showed an increasing correlation strength with time, as evidenced by strong positive values of *R^2^* = 0.7637 and 0.8155 at days 3 and 5, respectively. When the same analysis was applied to all proteins shared between these two cell types (*n* = 3148), the correlations only demonstrated a weakly positive (*R^2^* ≈ 0.13) relationship, indicating significant differences in the proteomes of OCs differentiated from RAW264.7 or BMM cells, as was observed in the hierarchical clustering analysis (Fig. 2 *C*).

### Cell cycle signaling, cytoskeletal organization, and apoptosis were significantly altered in RAW 264.7 cells as compared to BMMs

**Figure 3:**
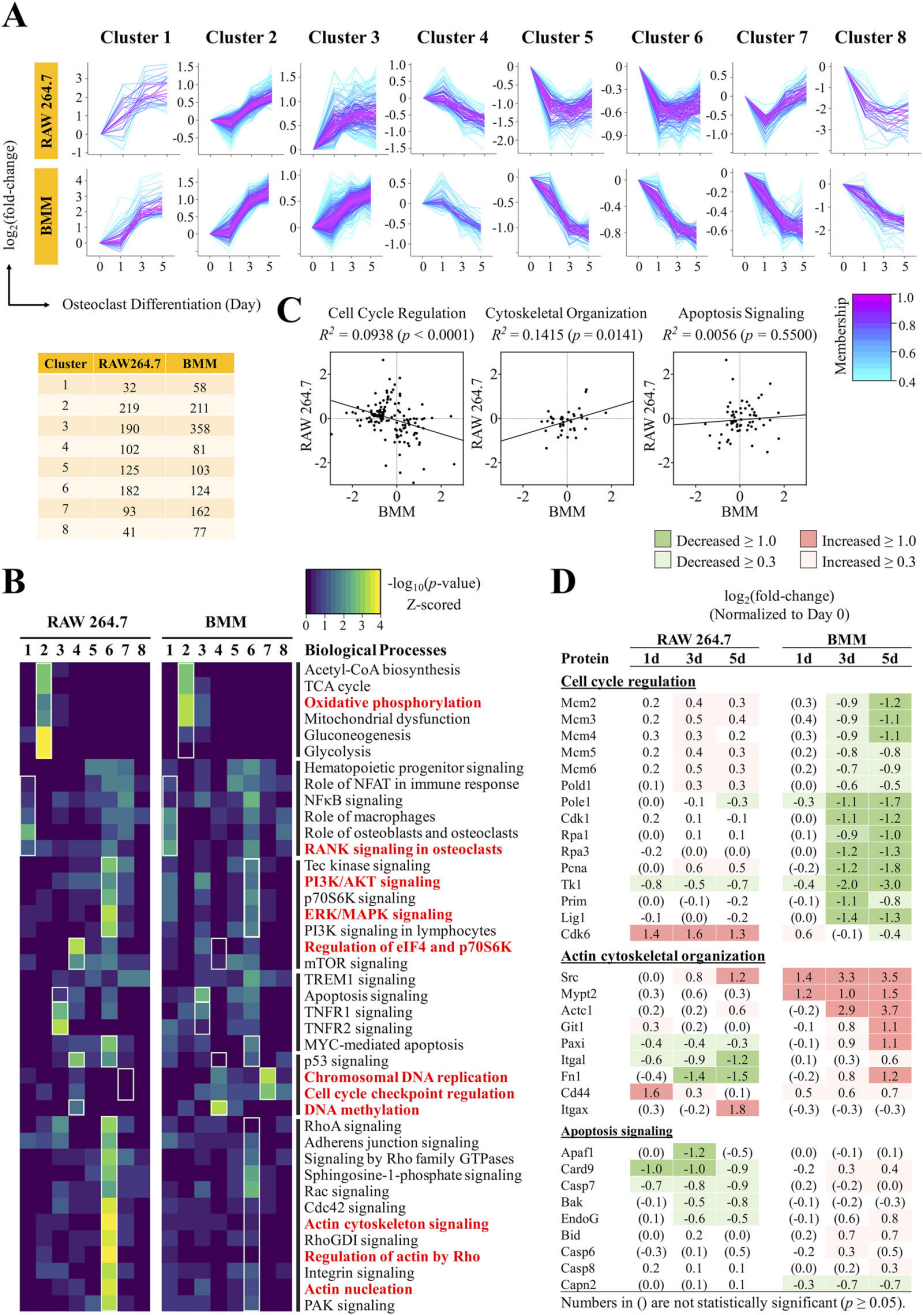
Comparative analysis of temporal expression profiles identified diverse biological processes significantly different between RAW 264.7 cells and BMMs. (*A*) Fuzzy c‐means clustering of significantly altered proteins to determine the temporal patterns that characterize the OC proteome. Membership scores indicate the degree to which proteins belong in each cluster. The number of proteins belonging to each cluster is indicated in the table below. (*B*) Gene ontology term analysis for biological processes that represent the proteins in each fuzzy c‐means cluster. Red bolded terms indicate biological processes that were strongly enriched and/or strongly contrasted between RAW 264.7 cells and BMMs. (*C*) Pearson correlation analysis applied to proteins involved in cell cycle signaling, cytoskeletal organization, and apoptosis signaling at day 5 of OC differentiation. (*D*) Table of key proteins found to be poorly or negatively correlated between RAW 264.7 cells and BMMs.

Given the weak correlation between the proteomes of OCs derived from RAW 264.7 cells and BMMs, we sought to identify the biological processes that were differentially regulated. We first characterized the temporal dynamics of the OC proteome, through examining the significantly altered proteins by fuzzy c‐means clustering (Fig. 3 *A*). The analysis revealed eight distinct patterns of protein expression, which were generally similar between RAW 264.7 cells and BMMs. The expression of these proteins was increased in clusters 1, 2, and 3; transiently increased in cluster 4; or decreased in clusters 5, 6, and 8. Although clusters 1, 2, and 3 all contained proteins with increased expression, the different clusters corresponded to different intensities of change. Cluster 1 encompassed proteins that were dramatically increased (2‐ to 4‐fold change), whereas cluster 2 contained proteins with moderate increases (∼1‐fold change), and cluster 3 with proteins that were slightly increased (∼0.5‐fold change). The same was observed for the downregulated clusters, where cluster 6 (and BMM cluster 7) represented slightly decreased proteins (∼0.7‐fold change), cluster 5 for moderately decreased proteins (∼1‐fold change), and cluster 8 for intensely decreased proteins (∼2‐fold change). In this analysis, we paired the clusters based on their direction and magnitude of change, but we identified a group of proteins in RAW 264.7 cells (cluster 7) whose temporal profile did not have a “mirror” in BMMs. These proteins were transiently decreased at day 1 but became slightly increased by days 3 and 5. Furthermore, although clusters 5, 6, and 8 in BMMs demonstrated a steady decline in protein expression at the later time points, the downregulated proteins in RAW 264.7 were transiently decreased at days 1 and 3, then reverted at day 5 (Fig. 3 *A*). We next performed GO term analysis to determine the biological processes associated with these temporal patterns (Fig. 3 *B*). Interestingly, clusters with similar trends and magnitudes of expression were involved in specific biological functions across both cell types. Cluster 1, for example, representing proteins that were strongly upregulated, was most closely involved in “RANK signaling in osteoclasts.” Cluster 2, representing the moderately upregulated proteins, was enriched in “energy metabolism” for both RAW 264.7 cells and BMMs. These observations indicate that the cellular abundance of proteins is tightly regulated during OC differentiation and may be characteristic of particular biological processes. The analysis also revealed several functional differences between the two cell types. First, proteins involved in cell cycle control were significantly overrepresented in BMMs (cluster 7, moderately decreased) but not in RAW 264.7 cells. Correlation analysis of protein expression related to cell cycle control (*n* = 162) indicated a weak but significant *negative* correlation between RAW 264.7 cells and BMMs at day 5 (*R^2^* = 0.0938) (Fig. 3 *C*). Closer inspection of these proteins showed that multiple components of the DNA replication complex, notably the members of DNA helicase complex (Mcm2‐6), family B DNA polymerases (Polδ1, and ε3), ssDNA‐stabilizing proteins (Rpa1/3), proliferating cell nuclear antigen (Pcna), and topoisomerases, were downregulated to varying degrees in BMMs at day 3 and day 5 (Fig. 3 *D*, Supplemental Fig. S1). In contrast, these proteins were either unchanged or upregulated in RAW264.7 cells. Moreover, cell division protein kinase 6 (Cdk6), an important regulator of G1 phase progression and G1/S transition of the cell cycle, was intensely upregulated at all time points of OC differentiation in RAW 264.7. In BMMs, Cdk6 was slightly increased at day 1 but was not significantly changed at days 3 or 5. Our data indicate that BMMs cease proliferation after the precursor cells have committed to OC differentiation by day 3. In comparison, RAW 264.7 cells actively proliferate even at the later stages of OC differentiation. This distinction was not unexpected because uncontrolled proliferation is a hallmark feature of transformed cells such as the RAW 264.7 cell line.

**Figure 4:**
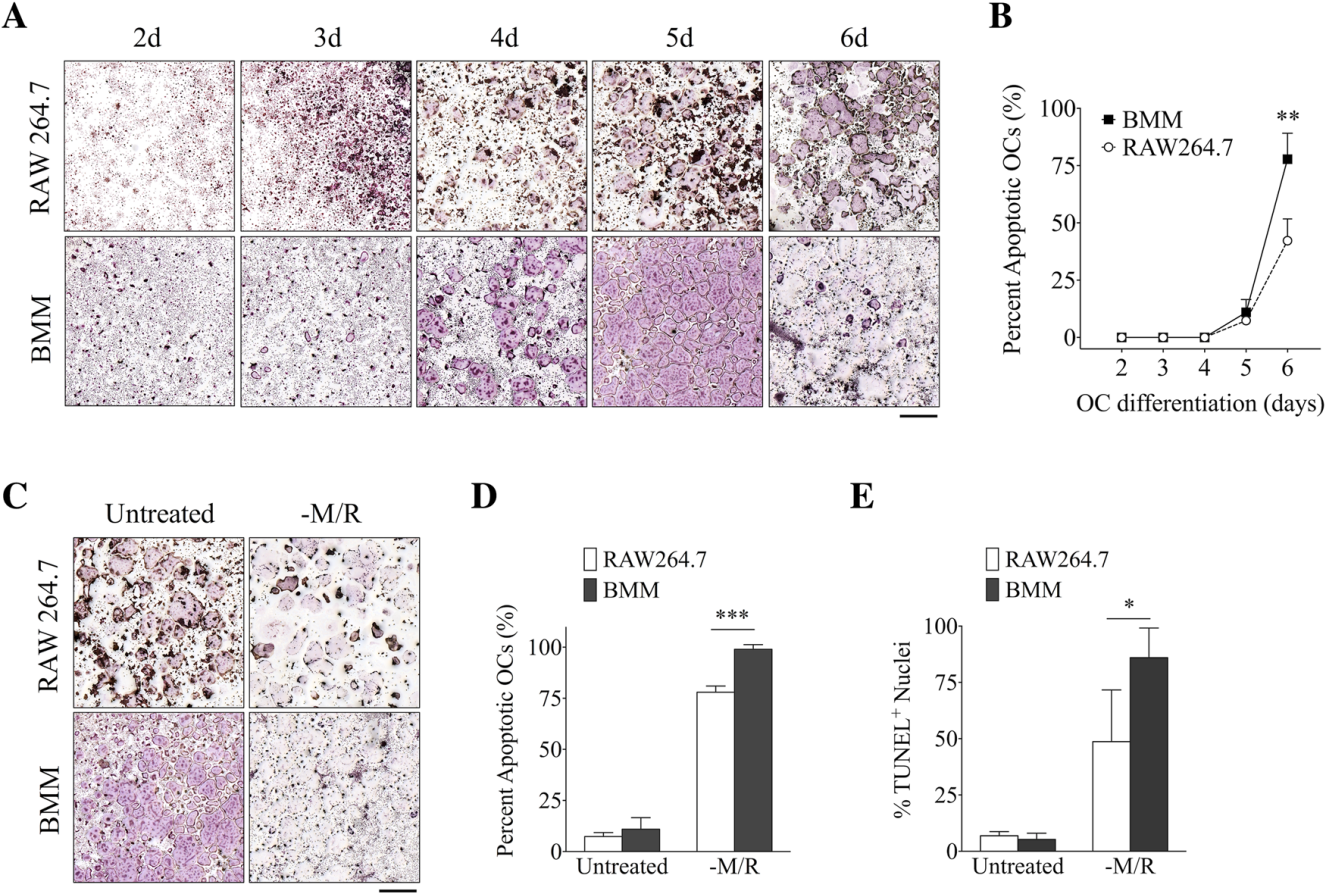
OCs derived from RAW 264.7 cells have extended life spans compared with BMMs. (*A*) TRAP staining of OCs derived from RAW 264.7 cells and BMMs induced with M‐CSF and RANKL for 2 to 6 days. (*B*) The number of dead OCs were quantified and presented as a percentage of the total number of OCs. (*C*, *D*) TRAP staining of mature OCs depleted of M‐CSF and RANKL for 16 hours. (*E*) Percentage of TUNEL^+^ nuclei in mature OCs depleted of M‐CSF and RANKL for 16 hours. Scale bar = 1 mm. Data are means ± SD. Statistical analysis was performed using Student's *t* test (*p < 0.05, ***p < 0.001).

OCs derived from BMMs and RAW 264.7 cells are capable of resorbing ivory or hydroxyapatite wafers *in vitro*, but we were interested in whether proteins related to the bone resorption process might be differentially regulated between the two cell types. The GO enrichment analysis revealed a significant overrepresentation of proteins involved in actin cytoskeleton signaling in RAW 264.7 cells (clusters 6 and 7) (Fig. 3 *B*), indicating that these processes were downregulated in RAW 264.7 cells during OC differentiation. In comparison, these processes were not as strongly enriched in BMMs (clusters 5 to 8). Closer inspection of these proteins revealed numerous inconsistencies in their expression patterns between these two cell types (Fig. 3 *D*, Supplemental Fig. S2). Correlation analysis of these proteins (*n* = 66) showed a poor correlation at day 5 (*R^2^* = 0.0145)—coefficients that are significantly lower than the global comparisons (*R^2^* ≈ 0.13) (Fig. 4 *C*). Notably, the proteins (e.g. c‐Src, α‐actinin, paxillin, cofilin‐2) that facilitate the formation of podosomes—actin‐rich, adhesive structures that form the sealing zone—were upregulated in BMMs during OC differentiation. These proteins, however, were not induced during OC differentiation in RAW 264.7 cells (Fig. 3 *D*, Supplemental Fig. S2). The data indicate a lower‐level expression of adhesion‐related proteins that might influence bone resorption in RAW 264.7 cells.

Because mature OCs are short‐lived, we asked whether OCs derived from RAW 264.7 cells, an immortalized cell line, have an extended life span. We therefore focused on this aspect in our analysis and found that apoptosis signaling was upregulated in BMMs (cluster 3), which was not observed in RAW 264.7 cells (Fig. 3 *B*). On the contrary, proteins related to “Myc‐mediated apoptosis” was downregulated in RAW 264.7 cells (cluster 6). Correlation analysis of these proteins revealed no correlation in the expression levels between RAW 264.7 cells and BMMs at day 5 (*R^2^* = 0.0056, *p* = 0.5500) (Fig. 3 *C*). In BMMs, the pro‐apoptotic factors, BH3 interacting‐domain death agonist (Bid), caspase recruitment domain‐containing protein 9 (Card9), and caspase‐6 and ‐8 (Casp6/8), were slightly upregulated at day 3 and sustained into day 5 (Fig. 3 *D*), suggesting that apoptosis signaling was initiated as early as day 3 of OC differentiation in BMMs. In contrast, the pro‐apoptotic regulators such as apoptosis protease activating factor 1 (Apaf1), Card9, and caspase‐7 (Casp7) were downregulated in RAW 264.7 cells during the later phase of OC differentiation. As such, our proteomics data suggest that apoptosis signaling is altered in OCs derived from the immortalized RAW 264.7 cell line compared with BMMs.

### Osteoclasts derived from RAW 264.7 cells have extended lifespans as compared to BMMs

To further confirm the above finding regarding apoptosis, we compared the life spans of OCs differentiated from RAW 264.7 cells and BMMs in vitro (Fig. 4 *A*, *B*). At the peak of OC differentiation (day 5), approximately 9.2 ± 3.8% of OCs have undergone apoptosis in both RAW 264.7 cells and BMMs. When the OCs were maintained for an additional 16 hours, 77.8 ± 11.4% OCs have undergone apoptosis in BMMs compared with the 42.2 ± 9.5% in RAW 264.7 cells. When the OCs were swapped into media without M‐CSF/RANKL and cultured for an additional 16 hours, almost all OCs from BMMs have undergone apoptosis (98.9 ± 2.3%), but 78.0 ± 3.0% of OCs differentiated from RAW 264.7 showed apoptotic morphology (Fig. 4 *C*, *D*). In either condition where M‐CSF/RANKL were maintained or removed, significantly more RAW 264.7‐derived OCs remained after 16 hours. Similar results were obtained when assessing the number of TUNEL^+^ nuclei in cells depleted of M‐CSF/RANKL (Fig. 4 *E*). This finding demonstrates that OCs from RAW 264.7 cells have an extended life span or are more resilient to apoptosis compared with OCs derived from BMMs.

### Abl is expressed in BMMs and RAW 264.7 cells and is required for osteoclastogenesis

**Figure 5:**
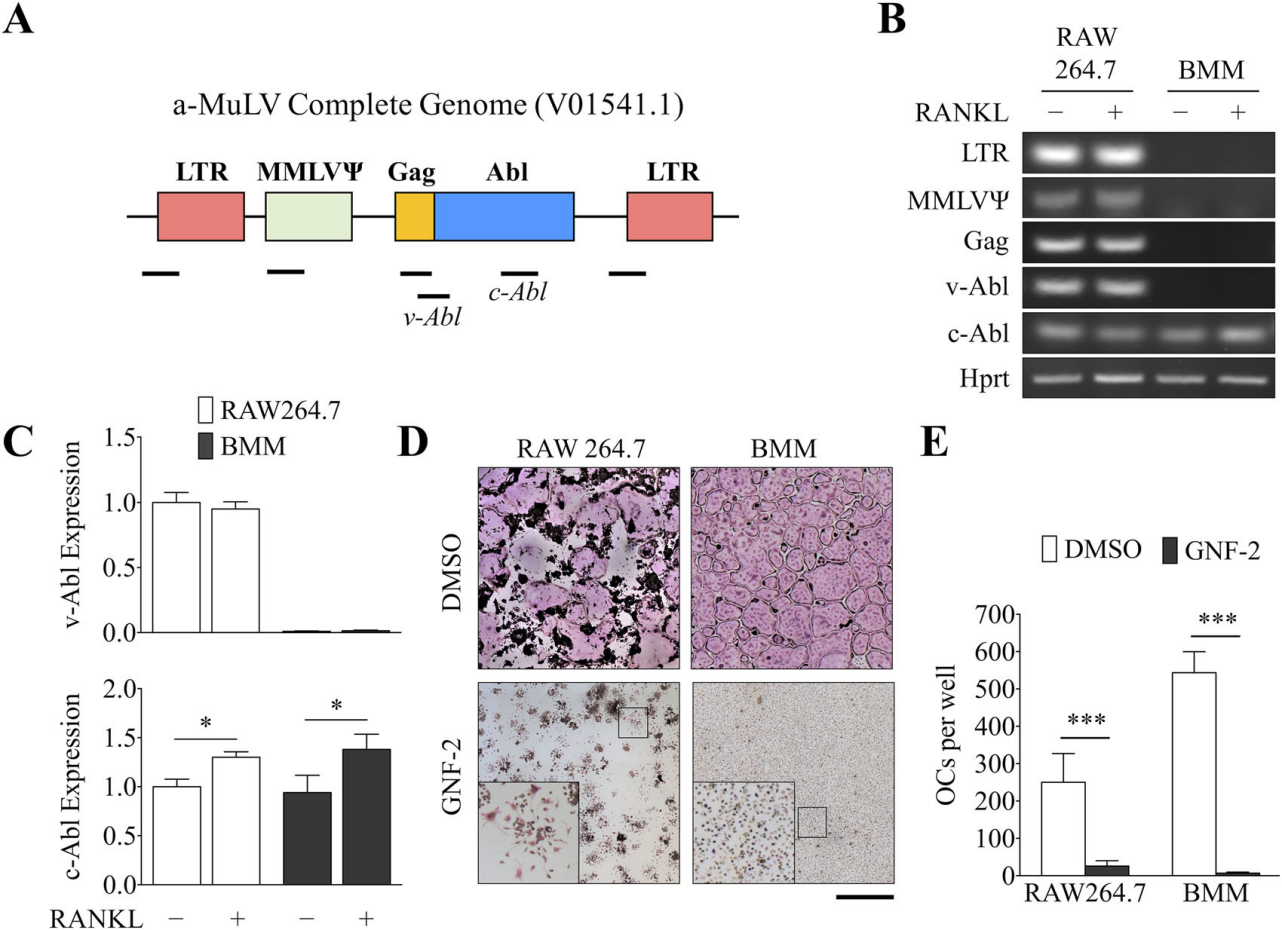
The viral oncogene v‐Abl is expressed in RAW 264.7 cells and Abl is required for osteoclastogenesis. (*A*) Diagram illustrating the structure of the integrated Abelson murine leukemia virus (a‐MuLV) genome and the relative regions amplified by RT‐PCR (black bars). LTR = long terminal repeats; MMLVΨ = Moloney murine leukemia virus Psi packaging element; Gag = group‐specific antigen; Abl = Abelson tyrosine kinase. (*B*) RT‐PCR was used to detect the various components of the a‐MuLV genome in RAW 264.7 cells and BMMs treated with or without M‐CSF/RANKL for 48 hours. (*C*) The relative expression of v‐ and c‐Abl were determined by qPCR and signals were normalized to *Hprt*. (*D*) TRAP staining of OCs treated with DMSO (vehicle) or the Abl inhibitor GNF‐2 (2 µM). (*E*) The number of TRAP^+^, multinucleated (>10 nuclei) cells were counted. Scale bar = 1 mm. Data are means ± SD. Statistical analysis was performed using Student's *t* test (*p < 0.05, **p < 0.01, ***p < 0.001).

We next sought to reconcile the observed functional differences with the fundamental property of RAW 264.7 cells—a cell line immortalized by the transforming activity of v‐Abl. Although the transforming activity of v‐Abl has been well characterized, it is not known how this constitutively activated tyrosine kinase affects OC differentiation and function. A previous study showed that GNF‐2, a selective inhibitor of Abl kinase activity, dose‐dependently inhibited the proliferation of OC precursors through the suppression of c‐Fms‐dependent signaling. GNF‐2 also accelerated OC apoptosis by inducing caspase‐3 and Bcl‐2‐like protein 11 (Bim) expression, interfered with actin cytoskeletal organization, and blocked the bone‐resorbing activity of mature OCs.^(43)^ Based on the consistency of our proteomics data to these findings, we predicted that altered Abl activity was likely responsible for the functional differences observed in RAW 264.7 cells. To test this hypothesis, we investigated whether the signaling downstream of Abl was regulated differently between these two cell types. RT‐PCR analysis using primers annealing to multiple regions of the a‐MuLV transcript confirmed that the viral transgene, v‐Abl, was specifically expressed in RAW 264.7 cells and not present in primary BMMs (Fig. 5 *A*, *B*). We also performed qPCR analysis to determine the relative expression of v‐ and c‐Abl. Primers that span the Gag and Abl domains are specific for v‐Abl. As was demonstrated by the RT‐PCR analysis, v‐Abl was only expressed in RAW 264.7 cells (Fig. 5 *C*). c‐Abl, on the other hand, was expressed in both cell types and was significantly upregulated by M‐CSF and RANKL (Fig. 5 *C*). In agreement with previous findings, GNF‐2 was a potent inhibitor of OC differentiation in both RAW 264.7 cells and BMMs, demonstrating that Abl is required for osteoclastogenesis (Fig. 5 *D*, *E*).

### Constitutive activation of Akt and Erk in RAW 264.7 cells are dependent on Abl

**Figure 6:**
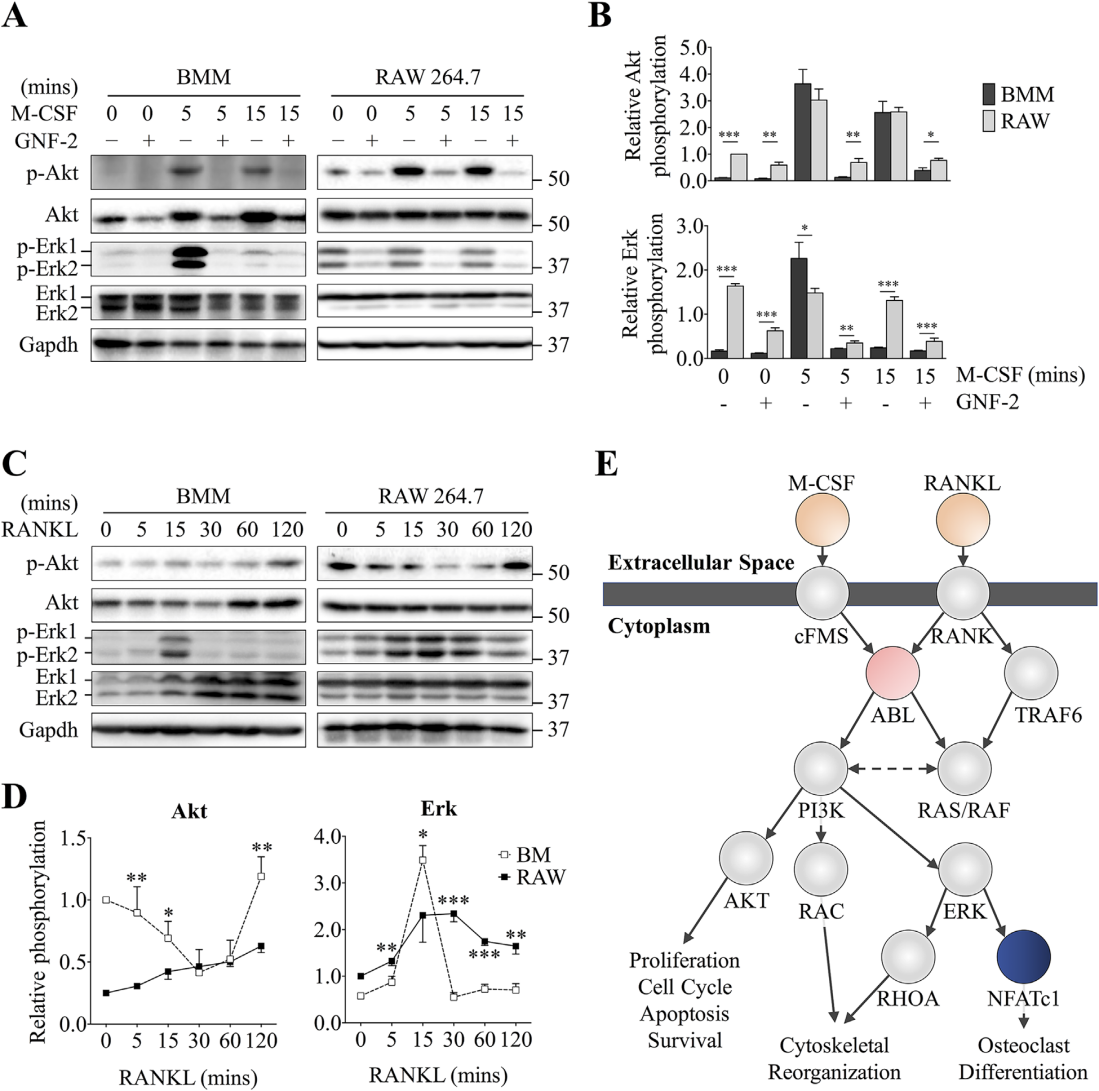
Constitutive activation of Akt and Erk1/2 in RAW 264.7 cells are dependent on Abl. (*A*, *B*) M‐CSF‐dependent phosphorylation of Akt and Erk1/2 in OC progenitor cells starved and pretreated with DMSO (vehicle) or GNF‐2 in serum‐free medium for 2 hours. Cells were treated with 100 ng/mL M‐CSF for the indicated times and analyzed by western blotting. (*C*, *D*) Time course of RANKL‐dependent phosphorylation of Akt and Erk1/2 in BMMs and RAW 264.7 cells serum‐starved and pretreated with DMSO or GNF‐2 for 3 hours and stimulated using 200 ng/mL RANKL for the indicated times. (*E*) Model of Abl‐dependent signaling pathway in OCs. Densitometry analysis was performed using ImageJ and normalized to the Gapdh intensity. Relative phosphorylation of Akt and Erk was presented as the ratio between the phosphorylated normalized to the nonphosphorylated/total protein. Data are means ± SD. Statistical analysis was performed using Student's *t* test (*p < 0.05, **p < 0.01 ***p < 0.001).

Because Abl was previously shown to regulate c‐Fms phosphorylation, we investigated whether M‐CSF‐dependent activation of Akt and Erk1/2 was altered in RAW 264.7 cells. BMMs and RAW 264.7 cells were serum‐starved and pretreated with GNF‐2 for 3 hours, then induced with M‐CSF for the indicated times (Fig. 6 *A*, *B*). Consistent with previous reports, M‐CSF treatment induced a potent and transient activation of Akt and Erk1/2 in BMMs (Fig. 6 *A*, *B*).^44^ In RAW 264.7 cells, Erk1/2 was phosphorylated even in the absence of M‐CSF, and the basal activation was sustained throughout the duration of the experiment. GNF‐2 inhibited the basal and induced phosphorylation of Erk1/2 (Fig. 6 *A*, *B*), suggesting that v‐Abl was responsible for the constitutive activation of Akt and Erk1/2 in RAW 264.7 cells. Interestingly, GNF‐2 treatment reduced the total abundance of Akt in BMMs but not in RAW 264.7 cells. Given that M‐CSF‐dependent signaling was regulated differently between RAW 264.7 cells and BMMs, we next determined how these cells respond to RANKL (Fig. 6 *C*, *D*). As before, the basal p‐Akt level in RAW 264.7 cells was relatively higher than that observed in BMMs. In response to RANKL, BMMs showed a gradual increase in Akt phosphorylation over time, whereas in RAW 264.7 cells, it was transiently decreased and reverted to original levels by 2 hours of induction (Fig. 6 *C*, *D*). Additionally, p‐Erk1/2 was sharply induced at 15 minutes of RANKL treatment but reverted to basal levels at 30 minutes and onwards in BMMs. In contrast, RAW 264.7 cells demonstrated a gradual induction of p‐Erk1/2 that reached its maximum at 30 minutes of RANKL treatment that slowly declined afterwards, and the rate of p‐Erk1/2 induction coincided with the decline of p‐Akt. Overall, the data indicated that the M‐CSF‐ and RANKL‐dependent phosphorylation of Akt and Erk1/2, effector molecules downstream of Abl, were differentially regulated between BMMs and RAW 264.7 cells (Fig. 6 *E*).

## Discussion

The RAW 264.7 cell line has undoubtedly been an important research tool in the study of OCs, in part because they are easy to handle and manipulate. Despite the extensive use of RAW 264.7 cells in our community over the past 18 years, surprisingly few studies have sought to characterize and compare them to the primary BMMs, to which they are supposed to model. This concern is especially disconcerting given that fundamental differences clearly exist, but no clear mechanism delineates the two cell models, and therefore it is not known whether OCs derived from RAW 264.7 cells can wholly replicate the behavior of the primary BMMs.

We employed a cutting‐edge proteomics strategy that pushes the boundaries of protein identification and quantification;^35^ consequently, our study provides the most comprehensive coverage of the OC proteome to date. In two most recent proteomics studies of OC differentiation from RAW 264.7 cells, one study identified 2068 nonredundant proteins (defined as ≥2 peptides per protein for identification with a 5% FDR)^29^ and the other identified 3729 unique proteins (≥2 peptides/protein and 1% FDR).^45^ In comparison, our study reliably identified 5566 unique proteins, a 49.2% increase in coverage, in RAW 264.7 cells using a similarly stringent criteria of ≥2 peptides per protein for identification with a 5% FDR (Fig. 1 *A*). Moreover, the majority of OC proteome studies have been confined to RAW 264.7 cells.^26^ To the best of our knowledge, our study is the first to investigate the proteome of OCs differentiated from BMMs,^26^, ^46^ which are often regarded to be more faithful to the mouse *in vivo* system compared with RAW 264.7 cells. Our proteomics analysis identified a total of 3498 unique proteins for BMMs. The lower number of proteins identified may be attributed to the heterogeneous nature of total bone marrow, which could result in fewer proteins that meet the peptides per protein identification criterion. Nonetheless, the proteomics data produced in this study will prove to be useful by updating the current OC proteome database with a substantially more comprehensive data set. Furthermore, the BMM‐based OC data set represents a rich trove of information for future data‐mining endeavors.

More importantly, this study outlines the first targeted and in‐depth analysis of differences between two prominent OC cell models from a molecular standpoint. A previous study compared the gene expression profiles of OCs derived from RAW 264.7 cells to that of primary cells and found that amongst the 405 genes used for the analysis, the two cell types expressed largely the same complement of genes (*R^2^* = 0.7599).^20^ In comparison, our correlation analysis determined a substantially lower correlation coefficient of *R^2^* = 0.1365 (day 5) (Fig. 2 *C*). Several plausible reasons might account this large discrepancy. First, the previous study used clonal populations of RAW 264.7 cells deemed to possess high osteoclastogenic potential; using purified precursor cells could enrich for genes related to OC differentiation. Second, numerous studies have consistently shown that gene expression levels do not correlate well with protein abundance; this phenomenon could therefore contribute to the discrepancy between the correlation analyses based on cDNA levels versus protein levels.^29^, ^40^, ^41^ In line with this thought, we assessed the protein levels of housekeeping genes reported to be stable at the transcript level in OCs and found that transcript and protein levels for some of these genes were inconsistent (Fig. 1 *D*).^34^ Third, the correlation analysis in the previous study analyzed only proteins strongly induced during OC differentiation (>100 fold‐change), potentially imparting selection bias to the results, whereas our analysis included the entire set of proteins identified. This phenomenon can be observed in the fuzzy c‐means clustering analysis, which showed that the most strongly upregulated proteins were strongly correlated between RAW 264.7 cells and BMMs, as these proteins were frequently involved in fundamental aspects of OC differentiation (Fig. 3 *A*, *B*). Similarly, when we performed the correlation analysis on proteins involved in the canonical signaling pathways of OC differentiation, which incidentally had the highest expression levels, the correlation coefficient reached as high as *R^2^* = 0.8155, bringing the score closer to that in the previous study (Fig. 2 *B*). Therefore, correlation analyses performed on a filtered subset of proteins could inadvertently produce skewed results, which is why we chose to perform the analysis on an unfiltered data set. Overall, our correlation analysis indicates that substantial inconsistencies exist within the proteomes of OCs derived from RAW 264.7 cells and BMMs.

Our comparative study unveiled a diverse set of biological processes that were differentially regulated between RAW 264.7 cells and BMMs during OC differentiation. Specifically, the regulation of proteins involved in cell cycle control, cytoskeleton reorganization, and apoptosis were significantly dissimilar. The coordinated actions of cell cycle withdrawal, initiation of differentiation‐associated gene expression, and cellular reorganization are key steps in the terminal differentiation of cells.^47^ In fact, studies have shown that OC progenitors withdraw from the cell cycle and are post‐mitotic at the time of fusion and terminal differentiation.^48^, ^49^ Consistent with previous findings, our results show that BMMs downregulated proteins involved with cell cycle control and chromosomal replication at days 3 and 5 of differentiation; RAW 264.7 cells, however, exhibited an opposite trend (Fig. 3 *C*). Notably, Cdk6 and Pcna were among the most highly upregulated proteins related to cell cycle control in RAW 264.7 cells. This observation does not, however, necessarily indicate that OCs from RAW 264.7 cells are actively proliferating at the terminal stages of differentiation. A previous study demonstrated that RANKL could dose‐dependently suppress proliferation in RAW 264.7 cells, likely because of cell cycle arrest caused by the downregulation of cyclin D1, D3, and E.^50^ Consistent with previous findings, our proteomics analysis found cyclin D1 to be strongly downregulated in RAW 264.7 cells during the course of OC differentiation (Fig. 3 *D*, Supplemental Fig. S1).

It has been reported that Abl plays a prominent role in regulating cytoskeletal rearrangement. Abl can coordinate changes to the cytoskeletal structure by regulating the activity of Rho family GTPases, stimulating the assembly of protein complexes that facilitate actin nucleation or directly interacting with F‐actin and microtubules.^51^ Moreover, the constitutive activation of Abl was shown to reduce paxillin phosphorylation and in turn reduce cell adhesion in murine fibroblasts.^52^ Interestingly, our result showed that a set of proteins involved in cell adhesion were upregulated in BMMs but not RAW 264.7 cells (Fig. 3 *D*, Supplemental Fig. S2). Additionally, paxillin expression was slightly downregulated in RAW 264.7 cells and upregulated in BMMs (Fig. 3 *D*). Therefore, our proteomics data indicate that RAW 264.7 cells have reduced expression of proteins involved in focal adhesion assembly and sealing zone formation.

Previous work has demonstrated a role for Abl in OC formation by regulating the phosphorylation of c‐Fms and the downstream effector molecules Erk and Akt.^53^, ^54^, ^55^ Conversely, GNF‐2, an inhibitor of Abl, suppressed M‐CSF‐dependent activation of Erk and Akt, inhibited the proliferation and differentiation of OCs, interfered with actin cytoskeletal organization, and accelerated apoptosis of mature OCs.^43^ Consistent with these reports, we found the aforementioned biological processes to be significantly altered in RAW 264.7 cells compared with BMMs (Fig. 5 *A*). Building on this hypothesis, we found that Abl‐related signaling through Erk and Akt showed a low yet constitutive activation in RAW 264.7 cells, and the temporal patterns of activation in response to M‐CSF and RANKL were distinct between these two cell types (Fig. 6 *A*, *B*). Despite the differences in Erk and Akt signaling in RAW 264.7 cells, our analysis demonstrated an exceptionally positive and linear correlation in the expression of RANKL‐dependent proteins (Fig. 2 *C*), indicating that RANKL‐dependent signaling was intact in RAW 264.7 cells. A recent study demonstrated that M‐CSF induced an immediate effect (5 to 20 minutes) on Erk phosphorylation, whereas RANKL triggered a biphasic activation—an immediate activation (5 to 20 minutes) and a delayed activation (8 to 24 hours). The second wave of Erk activation coincided with the onset of OC differentiation, indicated by the presence of TRAP^+^ mononuclear cells and the expression of marker proteins.^56^ Because we did not assess this aspect in our study, it is possible that the delayed activation was unaffected in RAW 264.7 cells, and therefore the cell line can still form OCs normally. The evidence from our study collectively indicates that the effects of v‐Abl in RAW 264.7 cells were limited to M‐CSF‐related signaling pathways. Therefore, our data suggest cautious consideration when using RAW 264.7 cells in future studies investigating signaling and processes downstream of M‐CSF.

Irregularities in RAW 264.7 cells, such as its independence from M‐CSF, are well‐known phenomena, but its effects on the practicality of the cell line as a model remained an important gap in knowledge. Our study indicates that the physiological effects of the transformed state on RAW 264.7 cells may be more significant than previously anticipated. Functions related to cell cycle control, cytoskeletal reorganization, and apoptosis signaling are likely affected in RAW 264.7 cells. Given the involvement of v‐Abl, other processes downstream of M‐CSF may also be implicated. In light of this, our proteomics data can be a valuable resource to other investigators to avoid potential pitfalls associated with RAW 264.7 cells.

## Disclosures

All authors state they have no conflict of interest.

## Supporting information

Supporting Data S1.
