# Supplementary material for: Comparative Characterization of Osteoclasts Derived From Murine Bone Marrow Macrophages and RAW 264.7 Cells Using Quantitative Proteomics
Source: JBMR Plus. 2018 Jul 7;2(6):328–40. doi: 10.1002/jbm4.10058 (PMC6237207; doi:10.1002/jbm4.10058)
Supplement: Supplementary file 1 — Supporting Data S1. [file JBM4-2-328-s001.docx]

**SUPPLEMENTAL METHODS**

**Isolation of Primary Bone Marrow Cells**

Bone marrow cells were obtained from the tibia and femur of 8-week-old C57BL/6J mice. Briefly, the cells were flushed from long bones, dispersed by pipetting, and cultured with complete α-MEM (Gibco BRL Invitrogen, Lawrenceville, GA, USA) containing 10% fetal bovine serum (VWR Life Science, Seradigm, Radnor, PA, USA), 12 mM _L_-glutamine and 100 U/mL penicillin and 100 μg/mL streptomycin (Gibco BRL Invitrogen, Lawrenceville, GA, USA)), and 20 ng/mL recombinant mouse M-CSF (R&D Systems, Minneapolis, MN, USA) for 24 hours. Nonadherent cells were re-seeded for subsequent experiments.

**Cell Culture and Osteoclast Differentiaition**

BMMs were seeded at 10,000 cell/mm2 and cultured for 2 days in the presence of 50 ng/mL M-CSF before inducing with 100 ng/mL RANKL for up to 5 days. RAW 264.7 cells were seeded at 70 cells/mm2 and stimulated with 50 ng/mL M-CSF and 100 ng/mL RANKL for up to 5 days. TRAP staining was performed using the acid phosphatase, leukocyte (TRAP) kit (Sigma-Aldrich, St. Louis, MO, USA). Cells were imaged using the Cytation 5 Cell Imaging Multi-Mode Reader (BioTek, Winooski, VT, USA) using the montage function where 8 × 6 frames (4× objective) were stitched together. OCs were quantified by counting the number of TRAP+, multinucleated cells (≥10 nuclei/cell) per well using the ImageJ cell counter function. TUNEL assay was performed using the TACS 2 Tdt-Fluor In Situ Apoptosis Detection Kit (Trevigen, Gaithersburg, MD).

**RT-PCR**

**Supplemental Table 1.** Primers used for the detection of v-Abl by RT-PCR.


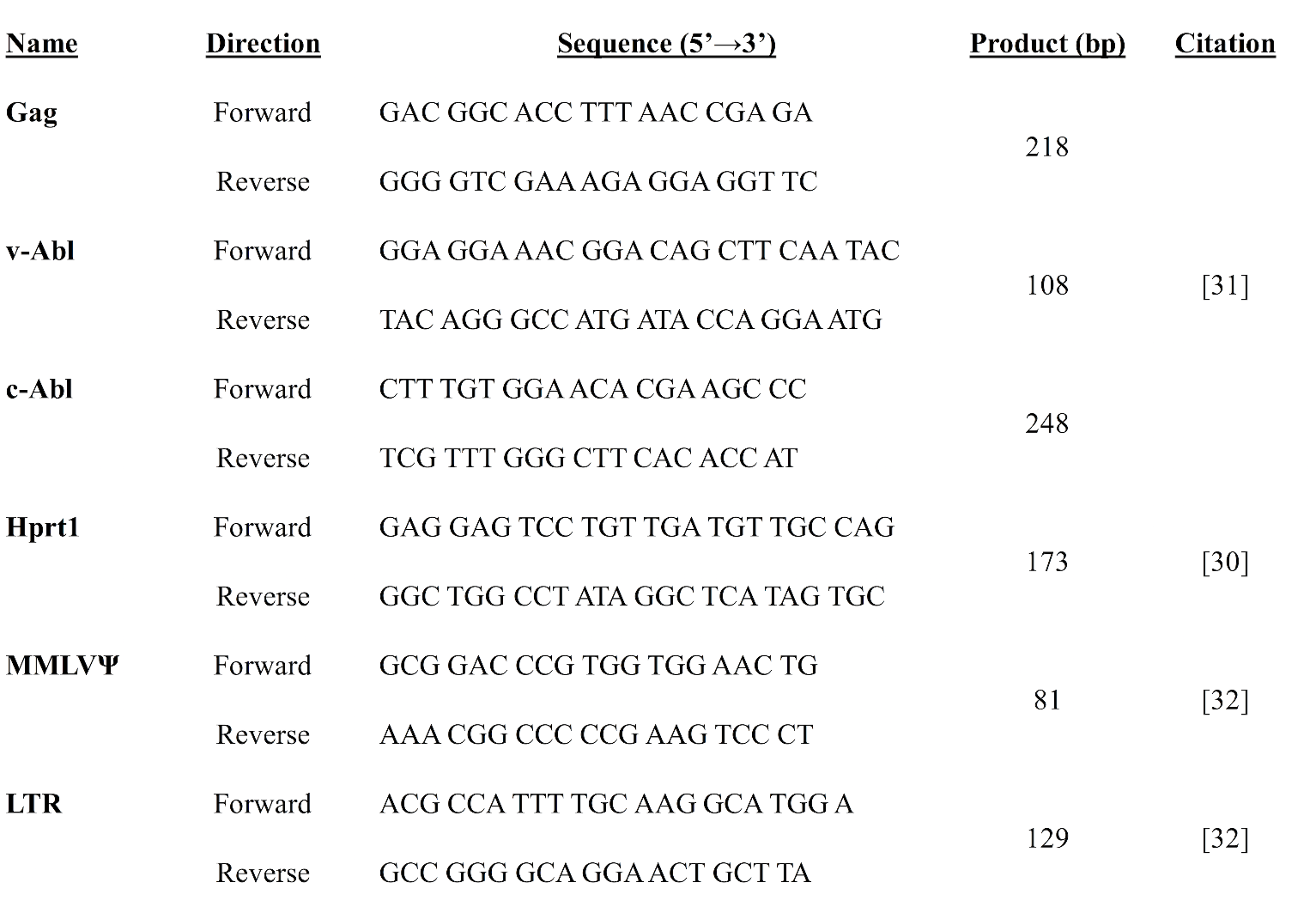


**Protein Extraction and Precipitation/ On-Pellet Digestion**

Cells were harvested using ice-cold lysis buffer (50 mM Tris-formic acid, 150 mM NaCl, 0.5% sodium deoxycholate, 1% SDS, 2% NP-40, pH 8.0) with protease inhibitor (cOmplete, Mini, EDTA-free; Roche, Mannheim, Germany). The cell lysate was sonicated for brief intervals (QSonica, Newtown, CT, USA) and centrifuged at 18,000 × *g* for 30 minutes at 4 °C. Samples containing 100 μg protein were reduced by the addition of 5 mM dithiothreitol for 30 minutes, followed by alkylation using 40 mM iodoacetamide for 30 minutes in darkness. The reduction and alkylation of proteins were performed at 37 °C with rigorous mixing at 200 RPM. The protein samples were precipitated by a stepwise addition of 6 volumes of chilled acetone with vortexing, and then were incubated at -20 °C for 16 hours. The protein was pelleted by centrifugation at 18,000 × *g* for 30 minutes at 4 °C. The pellets were washed using an 85/15% (v/v) chilled acetone/water solution, and then partially air-dried. The pellets were resuspended with 100 μL Tris-formic acid buffer (50 mM Tris, pH 8.5) and digested using trypsin at a 1:20 (w/w) enzyme/substrate ratio. The mixture was incubated at 37 °C for 6 hours with constant mixing at 200 RPM. The digestion was terminated by adding formic acid to a 1% (v/v) final concentration.

**Liquid Chromatography-Tandem Mass Spectrometry (LC-MS/MS) Analysis**

The peptide mixture was chromatographically separated by a nano-LC system (Eksigent, Dublin, CA) and analyzed using an Orbitrap Fusion mass spectrometer (Thermo Fisher Scientific, San Jose, CA). Mobile phase A and B were 0.1% formic acid in 2% acetonitrile and 0.1% formic acid in 88% acetonitrile, respectively. The nano-LC column was heated at 52°C to improve chromatographic resolution and reproducibility. Four μg of peptides were loaded onto a large-ID trap (300 μm ID × 0.5 cm packed with Zorbax 3 μm C18 material) with 1% B at a flow rate of 10 μL/min, and the trap was washed for 3 min. A series of nanoflow gradients (flow rate, 250 nL/min) was then used to back-flush the trapped peptides onto the nano-LC column (75 µm ID x 75 cm, packed with 3-µm particles) for separation. A 160-min gradient was applied in these analyses as previously described [30]. LC-MS/MS was acquired using data-dependent product ion mode for all samples. Capillary temperature was 250 °C. The instrument was run in top speed mode with a cycle time of 3 s. Monoisotopic precursor selection was used and peptide precursors with charge state 2–7 were sampled for MS2. MS1 survey scans (m/z range 400-1500) were performed at a resolution of 120,000 with an AGC target of 5 × 10^5^. MS2 was performed by isolation at 1.2 Th with the quadrupole for HCD fragmentation. The normalized collision energy was 35% with stepped collision energy of 5%. An activation q of 0.25 was used. Tandem mass spectra were analyzed by Orbitrap with a resolution of 15,000 in centroid mode, an AGC target of 5 × 10^4^, and max injection time of 50 ms. The dynamic exclusion was enabled with the following settings: repeat count, 1; repeat duration, 50 s; exclusion duration, 60 s; mass tolerance, ± 10 ppm.

**Western Blot**

Whole-cell extracts were prepared using radioimmunoprecipitation assay (RIPA) buffer (10 mM Tris-HCl, pH 8.0, 140 mM NaCl, 1 mM EDTA, 1% Triton X-100, 0.1% sodium deoxycholate, 0.1% SDS) with added protease and phosphatase inhibitors (Roche). Protein samples (50 μg) were resolved by SDS-PAGE using 10% Bis-Tris polyacrylamide gels and transferred onto PVDF membranes. Membranes were blocked at room temperature for 1 hour in Tris-buffered saline and 0.1% Tween 20 containing 5% bovine serum albumin. Incubation with primary antibodies was performed overnight at 4 °C followed by 1 hour of incubation with anti-rabbit horseradish peroxidase (HRP)-conjugated secondary antibodies (1:8000, Invitrogen). Membranes were developed using Clarity Western ECL substrate (Bio-Rad) and the protein bands were detected using the ChemiDoc MP imaging system (Bio-Rad).

**SUPPLEMENTAL DATA**


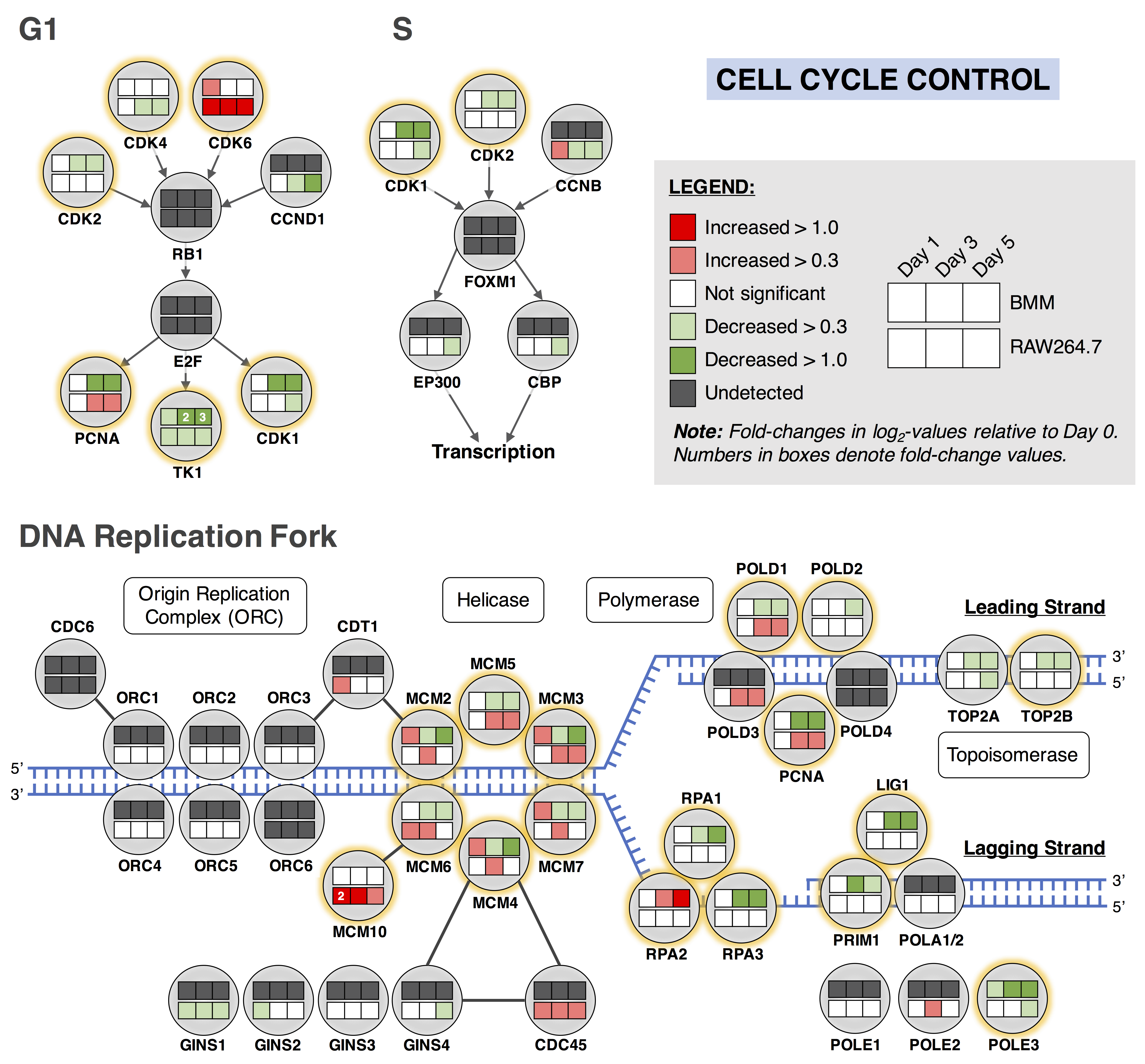


**Supplemental Figure 1.** Summary of proteins invovled in cell cycle control and DNA replication.


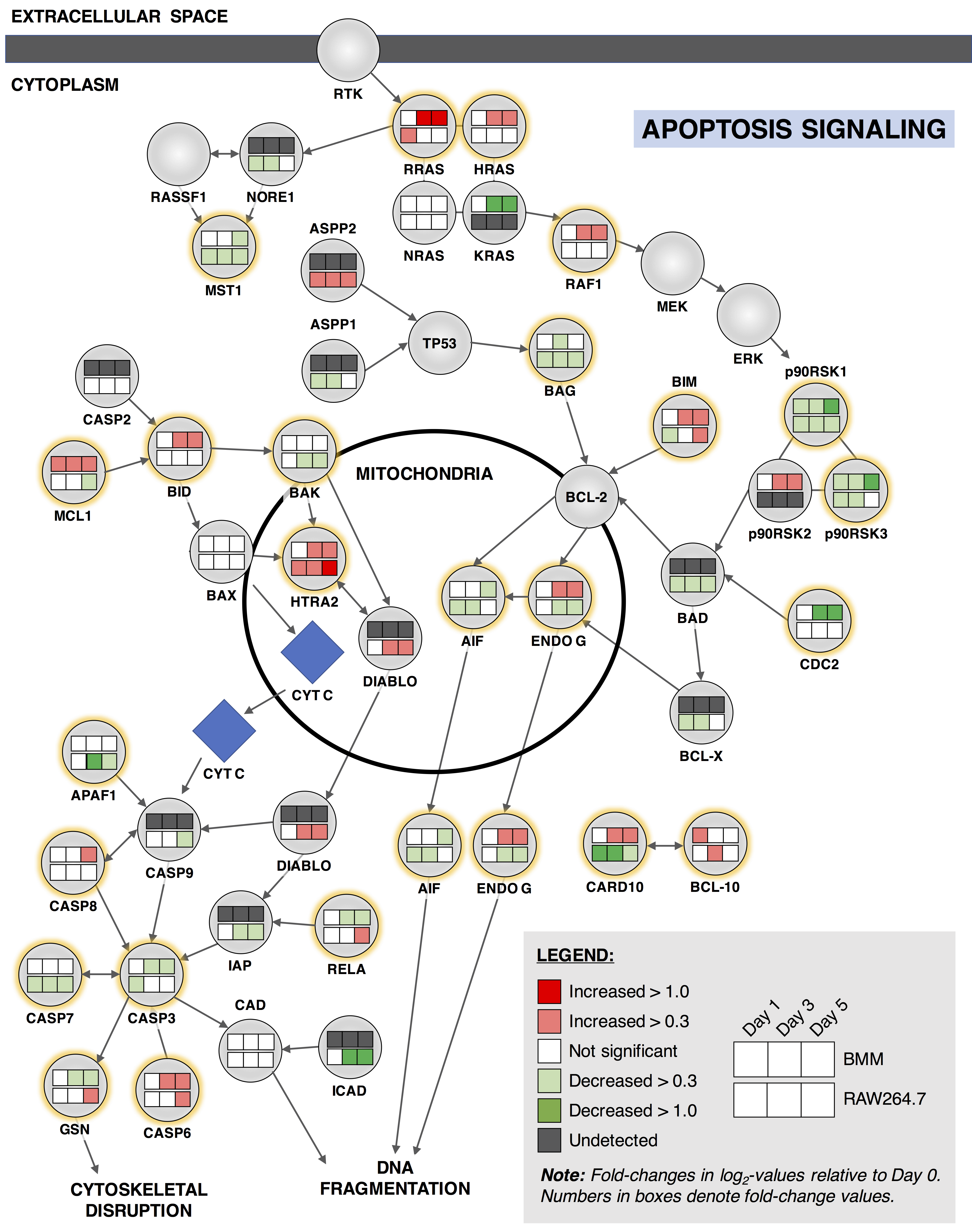


**Supplemental Figure 2.** Summary of proteins involved in apoptosis signaling.


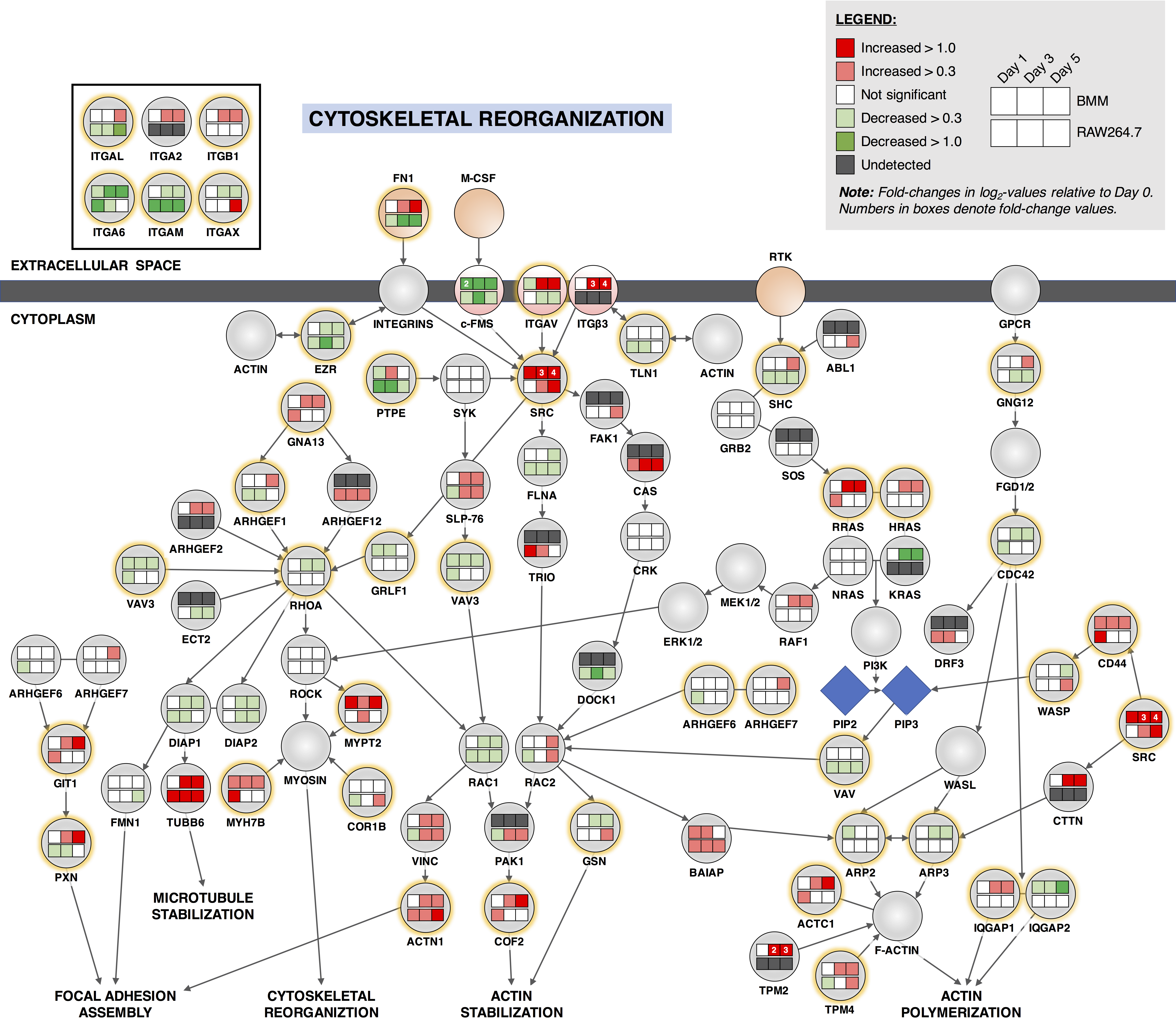


**Supplemental Figure 3.** Summary of proteins invovled in cytoskeletal reorganization.
